# Evaluating Autoencoders for Dimensionality Reduction of MRI-derived Radiomics and Classification of Malignant Brain Tumors

**DOI:** 10.1145/3603719.3603737

**Published:** 2023-08-27

**Authors:** Mikayla L. Biggs, Yaohua Wang, Neetu Soni, Sarv Priya, Girish Bathla, Guadalupe Canahuate

**Affiliations:** Foundation Medicine Cambridge, Massachusetts, USA; The University of Iowa Iowa City, Iowa, USA; University of Rochester Medical Center Rochester, New York, USA; The University of Iowa Iowa City, Iowa, USA; Mayo Clinic Rochester, Minnesota, USA; The University of Iowa Iowa City, Iowa, USA

**Keywords:** radiomics, dimensionality reduction, malignant brain tumors, autoencoders

## Abstract

Malignant brain tumors including parenchymal metastatic (MET) lesions, glioblastomas (GBM), and lymphomas (LYM) account for 29.7% of brain cancers. However, the characterization of these tumors from MRI imaging is difficult due to the similarity of their radiologically observed image features. Radiomics is the extraction of quantitative imaging features to characterize tumor intensity, shape, and texture. Applying machine learning over radiomic features could aid diagnostics by improving the classification of these common brain tumors. However, since the number of radiomic features is typically larger than the number of patients in the study, dimensionality reduction is needed to balance feature dimensionality and model complexity.

Autoencoders are a form of unsupervised representation learning that can be used for dimensionality reduction. It is similar to PCA but uses a more complex and non-linear model to learn a compact latent space. In this work, we examine the effectiveness of autoencoders for dimensionality reduction on the radiomic feature space of multiparametric MRI images and the classification of malignant brain tumors: GBM, LYM, and MET. We further aim to address the class imbalances imposed by the rarity of lymphomas by examining different approaches to increase overall predictive performance through multiclass decomposition strategies.

## 1 INTRODUCTION

Medical images collected from computed tomography (CT), magnetic resonance imaging (MRI), or positron emission tomography (PET) scans are increasingly used for the treatment and diagnosis of numerous diseases, including cancer. Radiomics is the extraction of quantitative imaging features to characterize tumor intensity, shape, and texture [9, 17, 19]. The primary benefits of quantitative radiomics are to generate meaningful, predictive image-based features that can be used in machine learning algorithms and precision medicine. There is a need for strong predictive modeling in cancer diagnostics and prognostics as early detection and proper treatment plans can be critical in a patient’s outcome.

Preoperative characterization of malignant brain tumors is critical in ensuring that patients receive the correct therapy as treatment strategies can differ. Parenchymal metastatic (MET) lesions, primary central nervous system lymphomas (LYM), and glioblastomas (GBM) make up the majority of malignant brain tumors in clinical neuro-oncology. LYM is a relatively rare form of brain cancer comprising only about 1.9% of all brain neoplasms. Gliomas are more common and account for approximately 80.8% of primary malignant brain tumors, with glioblastomas responsible for 57.3% of all gliomas. Generally, preoperative characterization is accomplished via MRI inspection and tumor resection or biopsy [20, 31]. While MRI imaging is a widely used imaging modality, these different brain tumors’ radiologically observed image features can be similar, making an observational characterization difficult. Likewise, tumor resection or biopsy is not error-free, with misdiagnosis and under-grading of tumors reported for 9.2% and 28% of neoplastic lesions, respectively [3]. Experts and physicians have modest success in characterizing these brain lesions; however, as imaging techniques advance, the volume of multiparametric MRI images grows, proving unreasonable for human interpretation. However, the growing imaging datasets prove ideal for artificial intelligence techniques such as feature engineering to improve the predictive power of the radiomic features. Machine learning and radiomics could aid in the classification of these common brain tumors and provide a more accurate preoperative diagnosis without additional invasive testing.

The number of radiomic features can range from hundreds to thousands and it is hard for physicians to select sets of features a priori because the exact biological correlates of these mathematical variables are yet to be established. Moreover, multiple features are often highly correlated, thus making it difficult to distinguish them. From a clinical perspective, large-scale validation of these features is more important than having physicians select a priori features when the exact biological correlates of such features remain unknown.

However, the large number of radiomic features extracted from these medical images and their associated high-dimensional feature space result in a common phenomenon in machine learning known as the curse of dimensionality. This curse of dimensionality arises when feature space becomes so large that the data is too sparse to accurately identify patterns and consequently diminishes predictive performance. In other words, a small increase in the feature space dimensionality requires an exponential increase in the volume of the input data to maintain similar performance. However, there are ways to combat this curse; dimensionality reduction is a feature engineering technique that can significantly reduce the number of features representing the high-dimensional radiomic space. The ultimate goal of dimensionality reduction in cancer research is to identify tumor signature profiles for cancer diagnosis and/or prognosis from the high-dimensional radiomic space.

Several techniques have been designed to reduce irrelevant and redundant features that burden machine learning tasks such as principal component analysis, factor analysis, and autoencoders in more recent years. Autoencoders are a form of unsupervised representation learning algorithms comprised of artificial neural network models designed to learn complex non-linear relationships to reconstruct the input data at the output. While with dimensionality reduction there is always some loss of information; autoencoders are attractive because with their non-linear activation functions they can obtain better characteristics and disregard unnecessary information and noise.

In this paper, we explore the effectiveness of autoencoders for dimensionality reduction on the radiomic feature space of multiparametric MRI images. Specifically, we investigate the effect of autoencoders on predictive performance for the identification of different brain tumors: GBM, LYM, and MET. We further address the class imbalance imposed by the rarity of lymphomas by examining different approaches to increase overall predictive performance, such as resampling and pipelining the classification tasks.

The rest of this paper is organized as follows. Section 2 presents related work. Section 3 describes the proposed approach and methodology used in this study. Section 4 presents and discusses the experimental results. Finally, Section 5 concludes this paper and presents directions for future work.

## 2 RELATED WORK

This section presents related work in the use of radiomics and dimensionality reduction in machine learning with a focus on the classification of malignant brain tumors and discusses multiclass decomposition as a method to address class imbalance.

Studies have shown that radiomics – the high-throughput extraction of image features from radiographic images holds great promise in medical imaging analysis [19, 32, 36]. Preoperative characterization of malignant tumors like LYM, GBM, and MET is often performed via MRI imaging and/or tumor biopsies. MRI-based radiomic features have been used for tumor classification of GBM, MET, and/or LYM [4, 18, 25, 26, 29, 35]. Most of these studies have done binary classification and only a few multiclass classifications.

Relevant to this work is the multiclass classification of the three most common malignant brain tumors: LYM, GBM, and MET using radiomic multiparametric MRI-image data [26]. The study compared the performance of twelve different ML models in combination with four feature extraction techniques. The study also tested the impact of the inclusion/exclusion of specific MRI image sequences, as with the two-class problems. The feature extraction methods evaluated included PCA [24], linear combination [27], a high correlation filter [10], and the embeddings generated by some machine learning models during training. The results showed that the T1-CE sequences perform well, but model performance varied significantly based on the features used for analysis, motivating the need for more robust feature extraction techniques in this space. The best-performing model was the generalized boosted regression model (GBRM) with embedded feature selection on the T1-CE sequences, though T1-CE alone performed similarly, achieving mean AUCs of 0.90 and 0.894, respectively.

In this paper, we examine feature extraction with autoencoders and its effect on the predictive performance of the three-class malignant brain tumor classification problem.

Autoencoders are a form of representation learning that effectively compresses the input data and ignores redundant information. They were initially introduced as a tool for feature extraction and dimensionality reduction but have been useful in generative modeling in recent years. The autoencoder architecture could be a simple feedforward network or a more complex architecture such as long-short-term memory (LSTM) networks [8, 22] and convolutional neural networks (CNNs) [2, 5, 13]. This extension of architectures allows for the application of autoencoders to more complex ML problems like anomaly detection and noise reduction.[1, 37].

Autoencoders have been successfully used in processing medical images. A study proposing convolutional autoencoders to extract features for identifying lung nodules from CT scans showed excellent performance showing that convolutional autoencoders can solve issues of time consumption and scarce labeling that come with ROI-based techniques [5].

Another study from Hoo-chang Shin applies stacked autoencoders for organ identification and visual and temporal feature extraction in 4D MRI images [28]. Stacked autoencoders are a deep learning architecture consisting of several layers of sparse autoencoders by stacking additional learning layers. An interesting architectural requirement of stacked autoencoders is that the number of nodes on either side of the bottleneck must be equal, i.e., mirrored. This study showed that all organ types were well detected by using stacked autoencoders for specialized feature extraction of temporal and visual features.

Though several machine learning techniques (MLT) were developed with binary classification problems in mind, these MLT can involve multiple classes. Moreover, a hypothesis is that the decomposition of multiclass problems into several binary classification problems could improve classification performance by allowing models to solve theoretically simpler tasks. A survey on the combination of binary classifiers for multiclass problems can be found in [21]. This review covers various decomposition strategies; the strategy employed in this work falls into the decomposition of a *k*-class multiclass problem into *l* binary classifiers where *l* is log_2_ *k*. Prior works show that decomposition strategies may reduce complexity in MLTs and improve class separation [7, 23]. One of the earlier applications of decomposition applies to digit identification where digits were linearly separable when considered in pairs of binary problems [16]. These binary results can then be combined to solve the multi-class problem. These decomposition strategies are twofold: the division of binary subtasks and strategies to combine the binary results. With prior studies showing good results in binary malignant brain tumor characterization, this work investigates decomposition strategies on the three-class problem by breaking the tumor characterization into two stages of binary MLTs.

## 3 PROPOSED APPROACH

**Figure 1:**
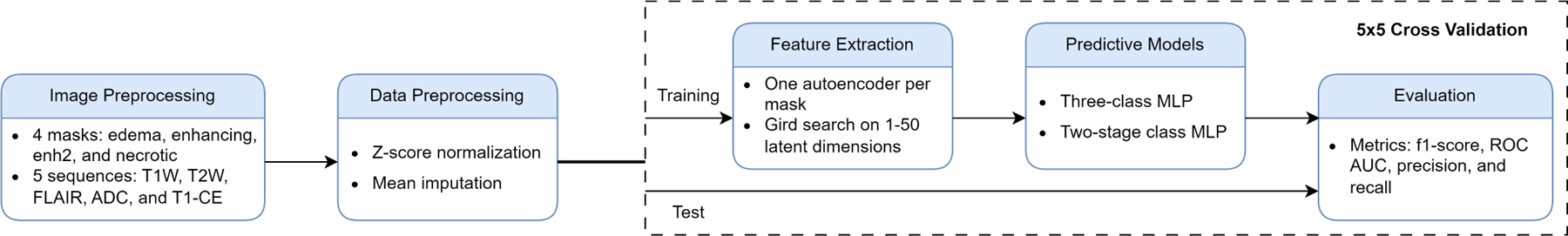
Project Overview, each box represents one major component of the proposed approach and serves as an outline for this work.

Figure 1 shows an overview of the methodology followed in this work. The first stage involves prior image processing work done by Dr. Bathla’s group to generate the four masks and five sequences that make up the multi-model MRI input data [26]. The output of this stage is the complete set of radiomics image features for the 20 image combinations. The second stage does the pre-processing to the full radiomic features including normalization and missing data imputation. In the feature extraction stage, different autoencoders learn the representation of the input image masks and choose an autoencoder with the best reconstruction error. With the reduced set of features, we then train a predictive model. Finally, we evaluate the performance of the models using cross-validation and different performance metrics. The rest of this section described the methods used in each stage in more detail.

### 3.1 Image Processing

MRI images were retrospectively collected for patients diagnosed with metastatic, glioblastoma, and lymphoma tumors. The multiparametric sequences, region-of-interests (ROI) masks, and the radiomic features were generated using the same processing pipeline as in previous work [26]. Specifically, preoperative imaging was performed to acquire five multiparametric sequences (T1W, T2W, FLAIR, ADC, and T1 contrast-enhanced) for each tumor instance and tumor segmentation was applied on each sequence to create four ROI masks (whole, enhancing, necrotic, and peritumoral edema), resulting in 20 mask-sequence combinations per input instance. Finally, the International Biomarker Standardization Initiative (ISBI) compliant radiomic features are extracted by Pyradiomics 3.0 on each of the combinations. The radiomic features include 3D shape, gray-level co-occurrence, and gray-level dependency matrix features, to name a few [34]^1^.

**Figure 2:**
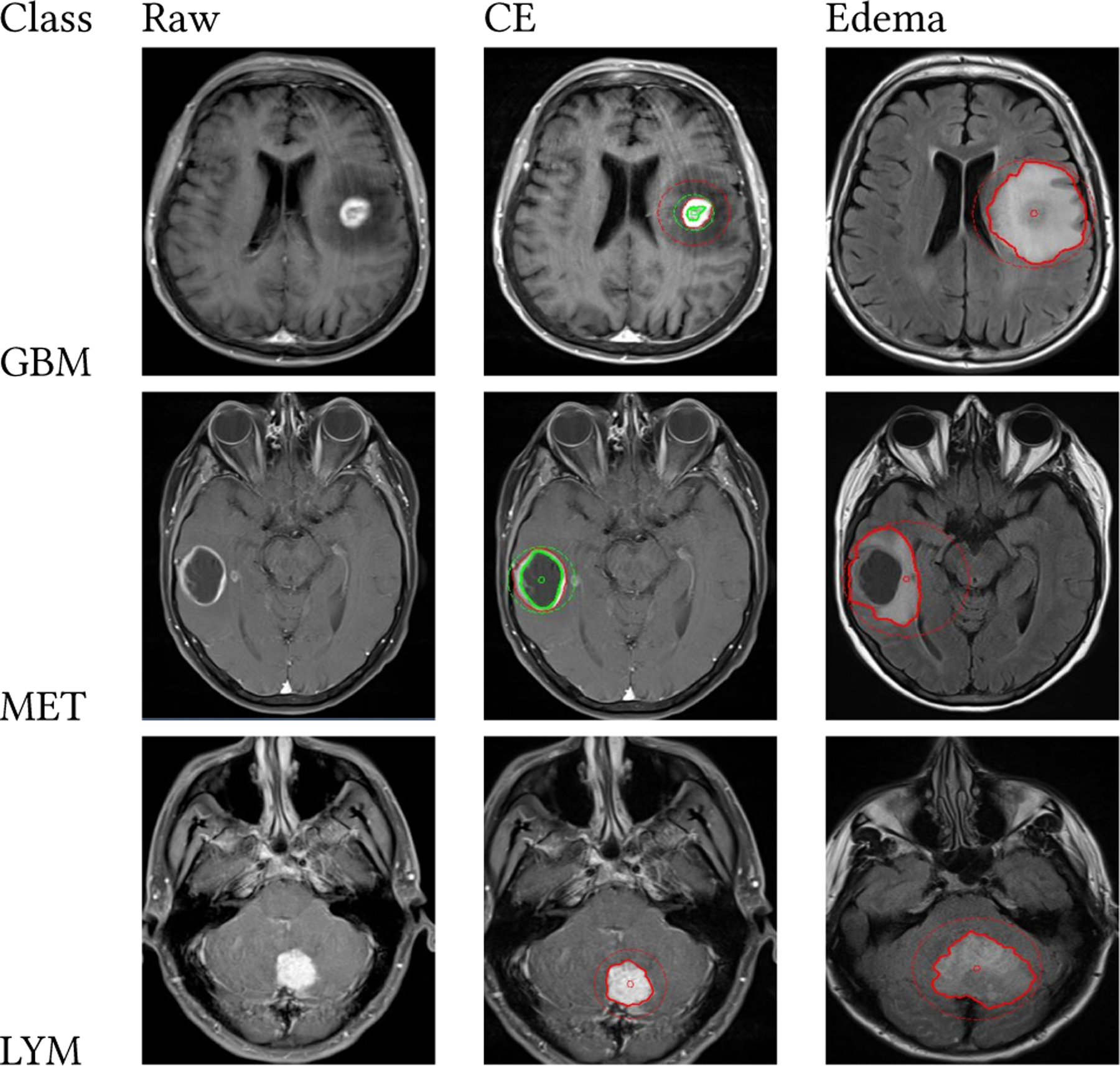
Example raw and contrast-enhanced MRI images and edema segmentation regions for each respective tumor class GBM, LYM, and MET.

Figure 2 shows examples of the raw MRI images, segmented contrast-enhanced sequences, and edema masks for each class. This figure illustrates how the different multiparametric MRI images and their associated ROIs look before radiomic feature extraction with Pyradiomics.

### 3.2 Data Preprocessing

Normalization and imputation were applied to the radiomic features as a pre-processing step before feature selection with the autoencoder. Normalization transforms the data to a common scale, ensuring that all features and instances similarly influence the model. We used standardization, i.e. z-score, for data normalization to transform the data features into a distribution with a 0 mean and a standard deviation of 1. Imputation was used to complete the missing data resulting from patients missing some masks or sequences. We decide to use mean imputation to conform to the approach followed in other related work [4, 25, 26]. Filling the missing values with zeros or a flag value of −9999 was also tested and did not differ much in reconstruction performance.

### 3.3 Feature Extraction

This feature learning stage uses the autoencoders to find a reduced set of features that represents the data and determine the number of latent variables needed to compress and represent the input data with an acceptable loss of information.

**Figure 3:**
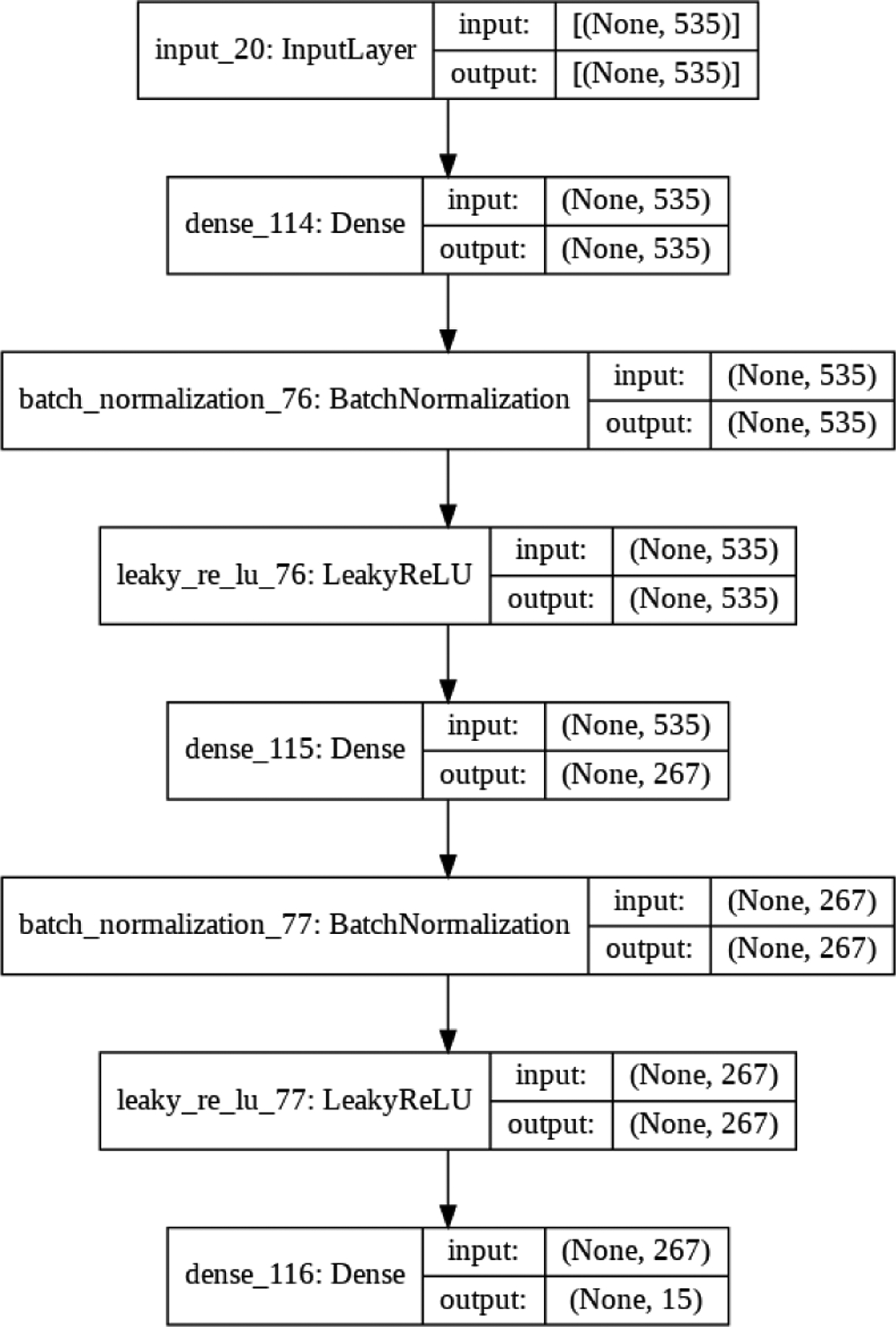
Encoder architecture visualization, specifying layers and nodes. Each box is a layer with “Dense” indicating fully-connected layers. The arrows indicate the flow of information between layers. The output is the compressed latent space representation of the input data.

One autoencoder is trained per mask. Figure 3 shows the architecture of the autoencoders used in this work. The autoencoder structure is a shallow ANN with one input layer followed by three fully connected (Dense) layers in the encoder and decoder (not shown in the figure) that mirror one another. The shape of the input is the number of features passed into the network with 535 being the number of features per ROI mask. The number of features is first halved to 267 and then compressed to the bottleneck dimensionality. The decoder takes the compressed representation, doubles the dimensionality, and reconstructs the data to the original shape. Reconstruction error was calculated using the mean-squared loss function and minimized with the Adam optimizer. The reconstruction error is used to determine the best latent size to represent each mask. The optimal number of latent variables could be different for each mask. We use a grid search to find the optimal latent space size by iteratively increasing the latent dimensions, ranging between 1 and 50. The training process stopping criteria is satisfied when the autoencoder is able to reconstruct the input data with a reconstruction error < 0.2.

The compressed representation learned from each mask is then concatenated as the feature vector to be used as input for the predictive models.

### 3.4 Predictive Models

Two model architectures were considered for the classification of MET, LYM, and GBM brain tumors: a three-class multilayer perceptron (MLP) model, and a two-level multiclass decomposition model that first train a binary classifier for the majority class, and then a second-classifier for the remaining two classes.

#### 3.4.1 Three-class Architecture.

Multiclass classification in machine learning is the problem of classifying instances into three or more target classes; this work implements a three-class classification task to predict GBM, LYM, and MET brain tumors using a MultiLayer Perceptron (MLP) model. The data is split into k-folds for validation with *k* − 1 training folds and one held-out test fold. The training begins by partitioning the training data by mask type, fitting an autoencoder to each, and saving the encoder with the lowest reconstruction error with early stopping as previously described. The final reduced set of features was then used to train the MLP model. For hyperparameter tuning, we implement an exhaustive parameter search using grid search to find the optimal parameters. Once the best-fit MLP learns the training data, the model predicts the held-out test fold. The predicted values are used to then evaluate the predictive performance of the trained MLP by comparing it to the ground truth labels. This process is repeated *n* = 5 times for *k* = 5 folds resulting in 5×5-fold cross-validation.

#### 3.4.2 Two-Stage Architecture.

The two-stage architecture aims to address class imbalance performance issues by breaking the three class problems into two, theoretically simpler, binary classification problems through multiclass decomposition. The first step is to divide the data into k-folds for cross-validation. The *k* − 1 training folds and then pass through two separate pipelines. The stage 1 pipeline addresses the first binary classification task to identify a primary target class, e.g. GBM yes/no. The stage 2 pipeline addresses the second binary classification task to distinguish between the two remaining secondary classes using the ground truth labels, e.g. MET/LYM.

As in the three-class classification task, an individual autoencoder is fit to each mask. Then, an MLP is fit to the re-coded compressed training data. The primary target samples are removed from the training folds, leaving only the secondary class’ samples. The minority class is then upsampled to achieve an equal distribution of classes in each fold to account for the class imbalance imposed by the rarity of LYM samples. Similar to stage 1, an autoencoder fits each mask, and a second MLP fits the stage 2 pipeline upsampled and encoded training folds.

The held-out test fold evaluates the predictive performance of stage 1 and stage 2 pipelines. The autoencoder is applied to the test set, and the MLP predicts the primary target class. The stage 2 pipeline validation differs slightly in that the test fold consists of all samples predicted as no for the primary class, e.g. “GBM” in stage 1. Any misclassified primary target sample will carry through into this test set; however, the predicted labels will be binary, motivating a need for an accurate stage 1 model. If a sample is misclassified in the first stage, it cannot be corrected in the second stage. The validation of stage 2 performance evaluates the binary predicted stage 2 labels versus the ground truth three-class labels. The test fold for stage 2 is not upsampled like in the training folds, and validation is performed on the true target class distribution. This results in an MLP predicting the primary target class, an MLP predicting the secondary class, and fitted autoencoders for the masks in each stage, ultimately creating a two-stage three-class predictive pipeline.

### 3.5 Validation and Evaluation

The validation method applied in this work is repeated stratified k-fold cross-validation (CV). Stratified k-fold CV aims to preserve the proportion of each target class in each fold using the ground truth labels. This procedure becomes repeated by performing the stratified k-fold CV *n* times. In this work, we consider repeated stratified k-fold cross-validation with *k* = 5 and *n* = 5. Stratifying each fold allows the model to train on a representative sample of data and is useful for imbalanced or small data sets like ours. Repeating the cross-validation tests different partitions of the k-folds and increases the generalizability. of the reported results.

To evaluate models’ predictive performance we report ROC AUC, precision, recall, and F1-score [12]. We do not report accuracy since it is not ideal for imbalanced class problems as correctly predicting only the majority class can give high accuracy scores with little learning. For multiclass problems, the AUC over all classes cannot be computed directly, but rather the multiclass AUC can be derived using the AUC per class by treating each as a binary classification problem. The most popular decomposition combination strategies for evaluating multi-class AUC are one-versus-one (OVO) [11] and one-versus-rest (OVR) [15]. OVO splits the data into *n* binary classification problems with one class versus every class, resulting in *n*(*n* − 1)/2 binary problems. OVR similarly split the data into binary classification problems with one per class resulting in *n* binary classification problems. We report the multiclass AUC using the OVR method.

Two kinds of averaging can be used to measure the performance for multiclass problems: micro and macro. The micro-average aggregates the contributions across all classes whereas the macro-average considers the measure for each class before averaging [30]. In other words, micro-average holds each *sample* with equal importance and macro-average holds each *class* with equal importance. Since we upsampled the minority class, there was little difference in reported measures for each averaging approach in our case so micro-averaging is used throughout.

## 4 EXPERIMENTS

In this section, we first describe the experimental setup and data used in this work. Then we present the results from the three-class and two-stage multiclass models.

### 4.1 Experimental Setup

The code was implemented in Python 3.7. The machine learning package used for the predictive models and model evaluation is sklearn (version 0.22.2). The machine libraries used to develop the autoencoder architecture were Keras (version 2.6.0), TensorFlow (version 2.6.0), and PyTorch (version 1.9.0). Additionally, NumPy (version 1.19.5) and pandas (version 1.1.5) were used for data modeling and analysis. In addition, other modules used include scipy (version 1.4.1) for standardization, and matplotlib (version 3.2.2) plus seaborn (version 0.11.2) for visualizations.

### 4.2 Data

The data used in this work correspond to 253 patients from a retrospective cancer study. The class distribution is presented in Table 1. There were 120 metastatic (MET), 93 glioblastoma (GBM), and 40 lymphoma (LYM) tumor instances. Pyradiomics 3.0 was used to extract 107 from each one of the 20 mask-sequence combinations from 4 masks (whole, enhancing, necrotic, and edema) and 5 sequences (T1W, T2W, FLAIR, ADC, and T1 contrast-enhanced). A total of 2,140 radiomic features per instance were extracted, which is a considerably large feature space. The full radiomic feature set was processed by mask, which resulted in four data sets with 535 radiomic features each.

### 4.3 Feature Extraction

**Figure 4:**
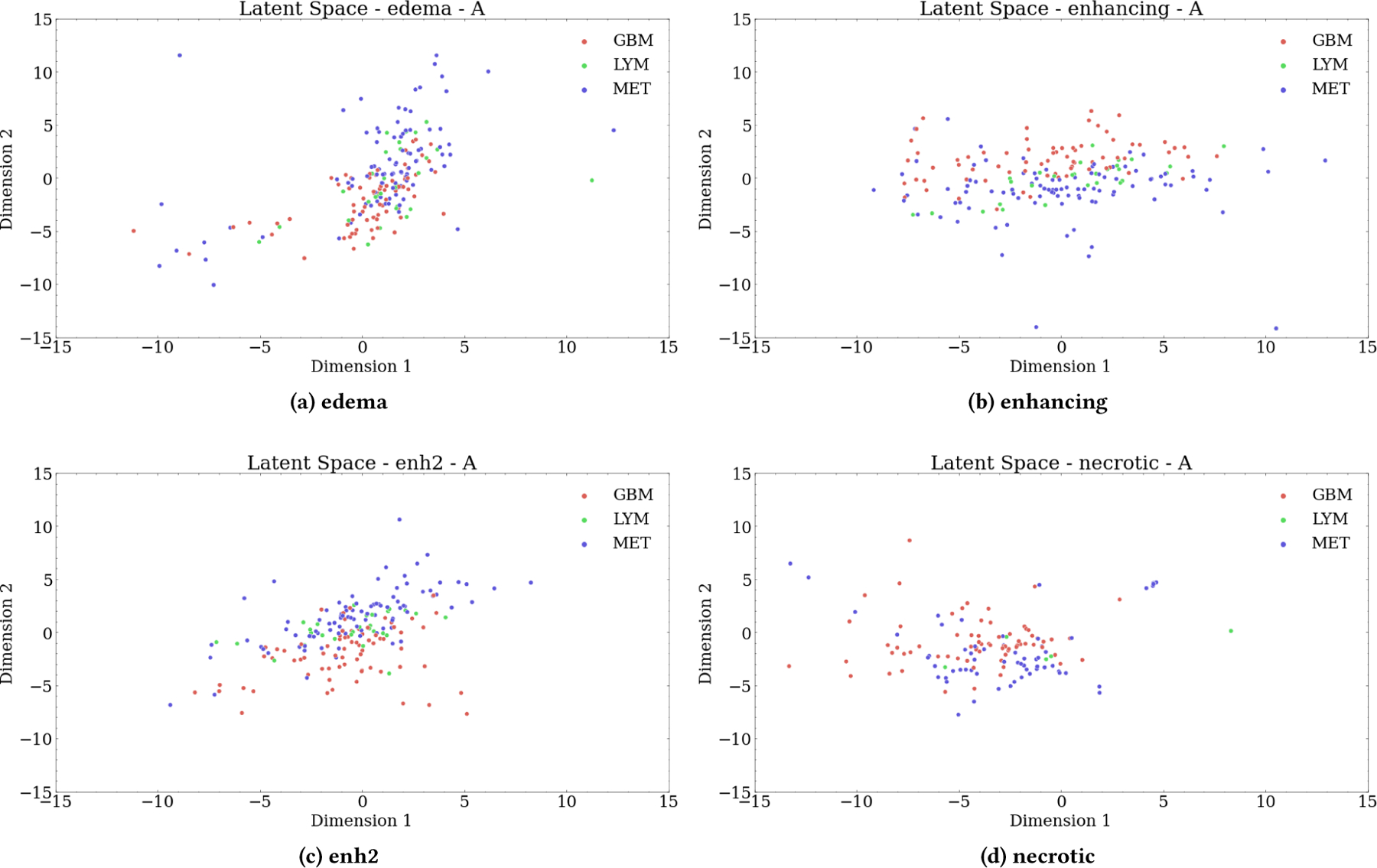
2-dimensional Latent Space per Mask for ADC Sequence

**Figure 5:**
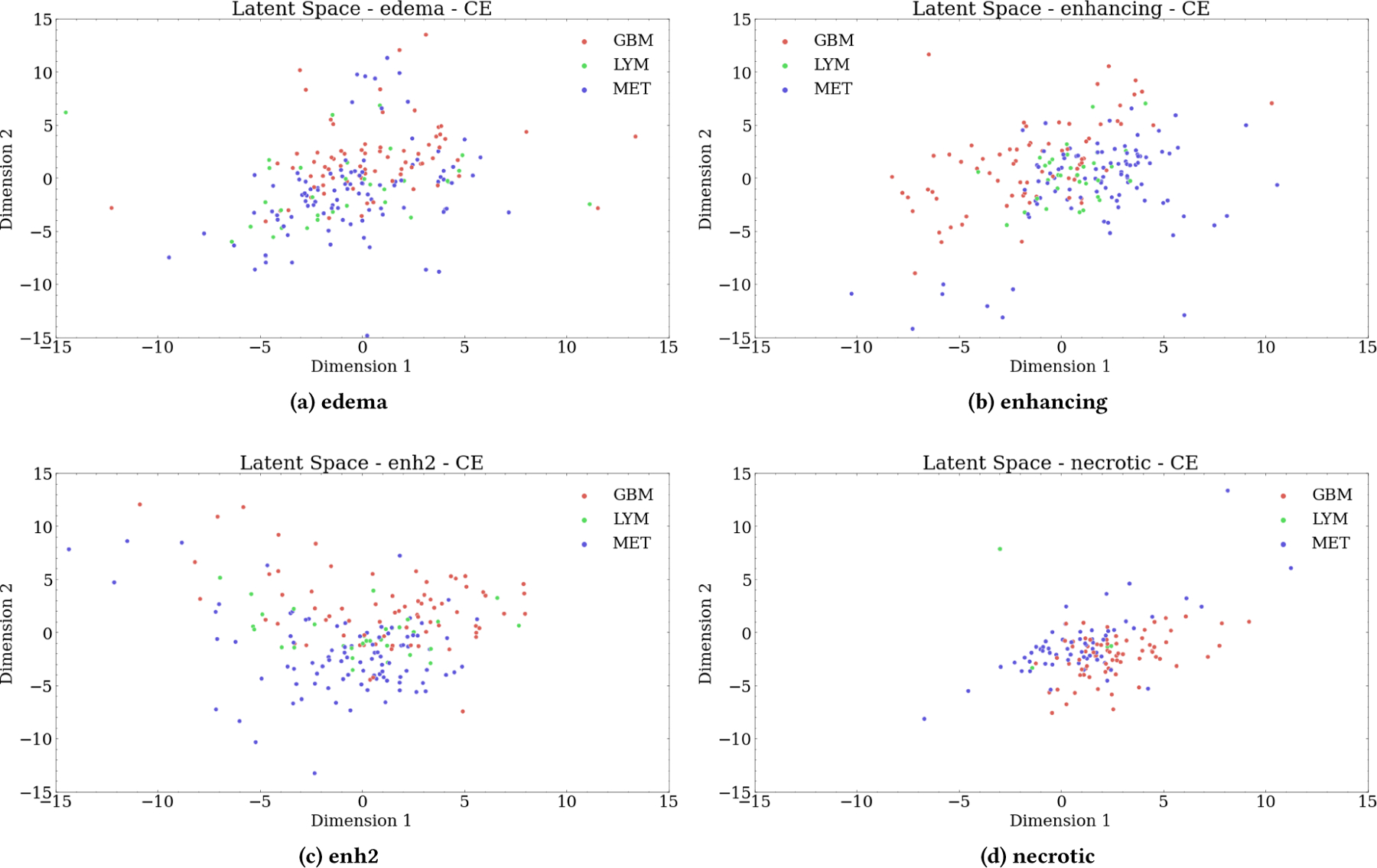
2-dimensional Latent Space per Mask for CE Sequence

To first evaluate the autoencoders’ potential, we generate a scatter plot for the 2-dimensional latent space for each mask/sequence independently. Figures 4 and 5 show the scatter plot for the ADC and CE sequences. The goal of these figures is to help visualize the separability of the tumor classes using the extracted features. The plot boundary for all these visualizations was set to [−15, 15] to ease the interpretation of the figures, but the latent space range is larger in some cases due to what seems to be outlier mappings. Figure 4 depicts the representation coding, or manifold, for each mask using only the ADC sequence. In all of [a–d], there is some separation between the GBM (red) and MET (blue) tumor classes, even with only two dimensions. However, there is no discernible separability for LYM in any manifolds, likely due to the class imbalance and imputed values mapping to a singular point. The separation in necrotic could again be due to the high volume of missing data within the necrotic masks. Figure 5 is the representation coding, or manifold, for each mask using only the CE sequence. Similar to the ADC manifolds, there is identifiable separability between GBM and MET but no real clustering in LYM, though the latent space for enhancing the CE sequence shows the best clustering of LYM in the center of the manifold. These 2-dimensional latent spaces are a minimal representation for each mask-sequence combination but that they are still able to capture enough information to show some separability between the classes is encouraging.

Our next task is to determine a suitable number of latent variables for each mask data set to reconstruct at least 80% of the data consistently, i.e., a reconstruction error < 0.20. An individual autoencoder was fit to each set and tested on latent sizes between 1 and 50. All autoencoders used in this work follow the structure outlined in the validation part of Figure 1, except the number of nodes in the central hidden layer was tuned to minimize error and dimensionality. Each autoencoder was trained for 250 epochs with early stopping ending when reconstruction error ceased to improve.

**Figure 6:**
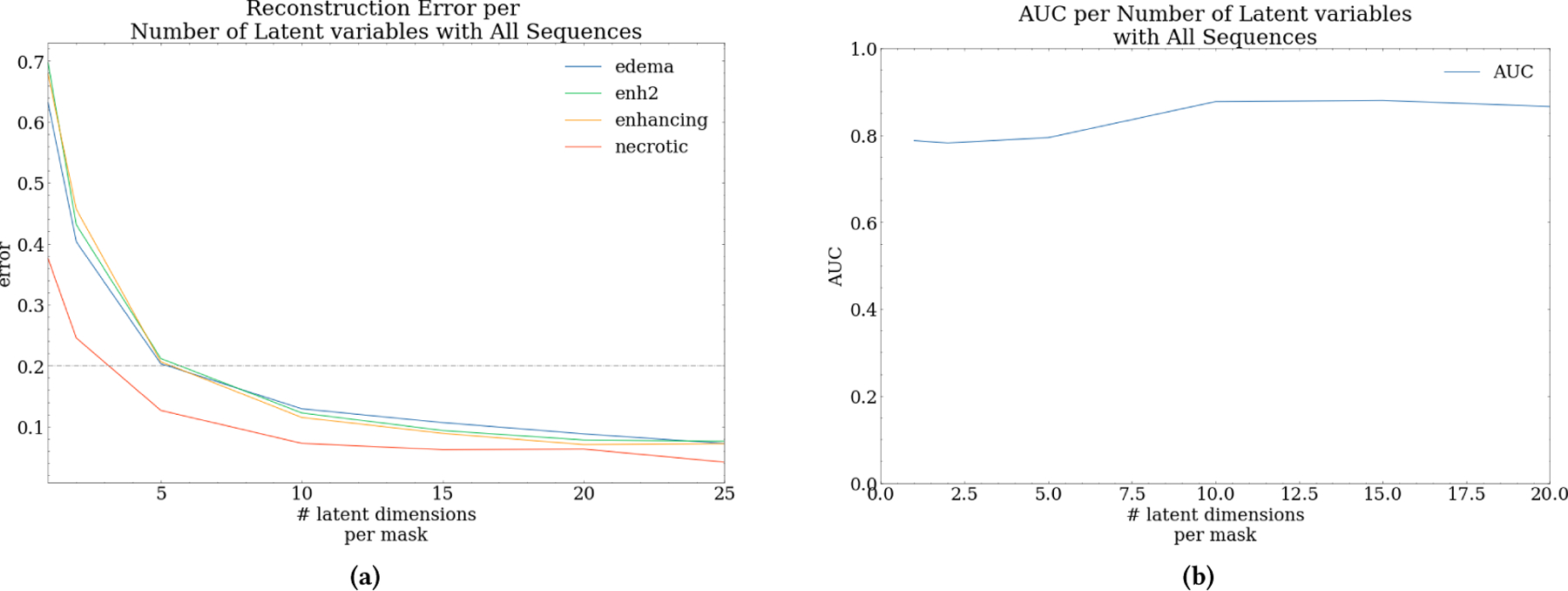
Evaluation of varying latent dimensions on reconstruction error and predictive performance of the multiclass MLP. (a) shows the reconstruction error as the latent dimension increases. (b) shows AUC as latent dimension increases

Figure 6a shows the reconstruction error results for latent sizes 1–25. For all four ROI masks, the reconstruction error rapidly converges below threshold 0.2 with as little as five latent variables. The loss curve for necrotic is significantly lower than the rest of the masks, likely due to necrotic containing the most missing values leading to the redundant reconstruction of the mean imputed values. From this figure, the range of latent variables sufficient to reconstruct the radiomic features can be hypothesized as 5 to 20 features per mask. The next step to narrow this range to an optimal latent size was to test the predictive performance of these representations. The predictive performance of the three-class MLP model is shown in Figure 6b across the latent variable range we determined. The overall trend of AUC is positive as the latent size increases; however, after around 15 latent variables, AUC begins to trend downward. A relatively similar performance is achieved starting at five latent variables and falling off around 15, supporting the hypotheses drawn from Figure 6a. The trend in AUC suggests that there is a window of performance in which there are enough latent variables to sufficiently represent the input and where the increase in latent size begins to introduce more error into the model.

### 4.4 Predictive Models

All of the predictive models applied in this work utilize hyperparameter tuning to improve classification performance. An exhaustive grid search of potential MLP parameters was run to determine the best combination of parameters like batch and hidden layer sizes. The sets of all values tested and the best parameters selected for each predictive model are shown in Table 2.

A shallow range of hidden layers was tested as deeper models would be too complex on our small class-imbalanced data. Likewise, a wide range of batch sizes was selected for testing with the class imbalance in mind hypothesizing that the smaller batch size makes the models less inclined towards the over-sampled majority classes and more discriminative to the under-sampled minority classes [12]. The number of all majority class batches would be more frequent and therefore have less pull on the model than the small batches that have both classes represented. Large values are used for batch size in some machine-learning problems to ensure that each batch captures positive samples. However, in our case, using a large batch size causes the activation from the sparse minority class to be averaged over time, diminishing its impact on model learning and resulting in a loss of performance. A small batch size ensures that the batches containing minority class samples have more discriminative weight on the model. An interesting result of the hyperparameter sweep is that the hidden layer sizes chosen reflect the complexity of the problem being solved, in this case. The more complex multiclass MLP needed more hidden layers to solve the three-class problem whereas the theoretically simpler binary classifiers only needed one.

#### 4.4.1 Three-class MLP.

**Figure 7:**
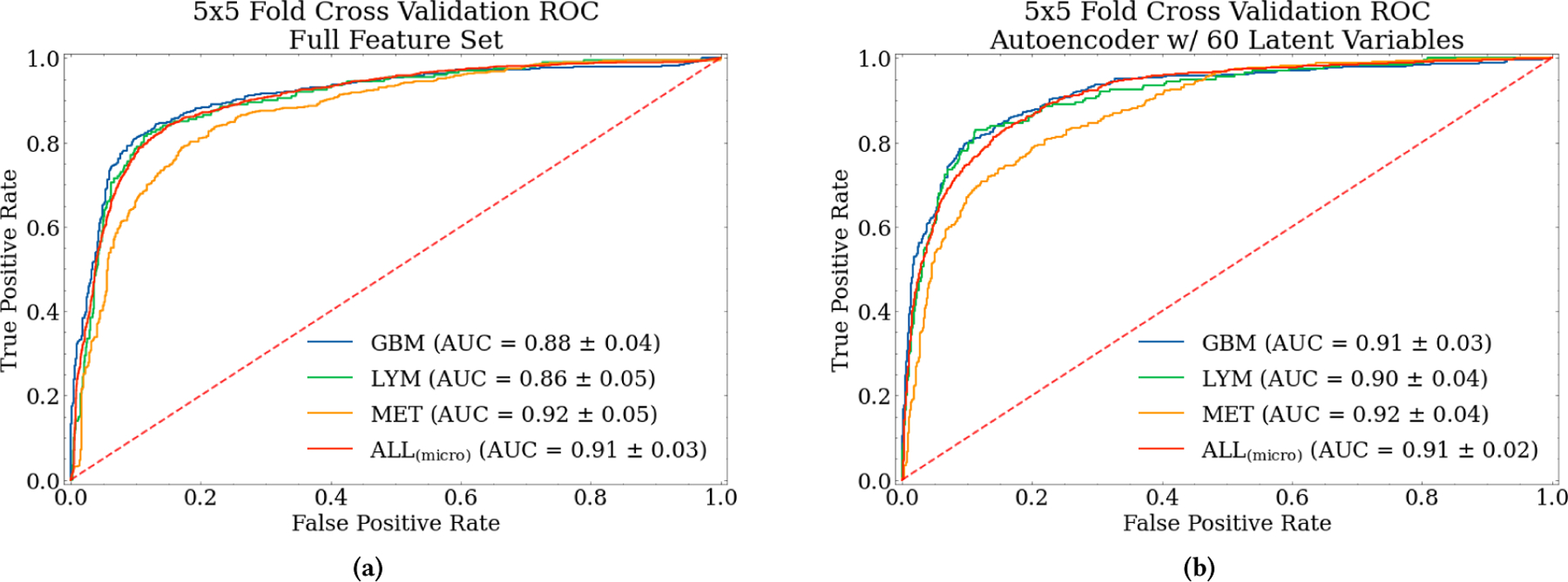
ROC AUC Curves for Three-Class Architecture. The full feature set (a) contains all the original radiomic features. The autoencoder model (b) contains 15 extracted features per mask, totaling 60 extracted features. The x-axis True Positive Rate is the probability of detection (sensitivity) and FPR is the probability of correctly predicting “other” (specificity). The dotted line indicates a random guess model.

The three-class predictive model was tested on the complete mask-sequence data and compared with the best autoencoder feature extraction results. The best autoencoder results were achieved using 15 latent variables per ROI in combination with the hyperparameters chosen in Table 2. The three-class model was fit to the complete data set as well as the best-performing autoencoder feature set. The AUC performance for each classification task is shown in Figures 7a and 7b, respectively.

We compare the performance of the feature extraction with the same model trained using the full set of features. The model achieved a 5×5-fold cross-validated AUC of 0.91. Under the same conditions, the extracted autoencoder features achieved the same AUC performance. The cross-validated performance of GBM and LYM classes shows promising performance improvements from the use of autoencoders. This slight improvement suggests that autoencoders could extract meaningful relationships from radiomic features while significantly reducing the high-dimensional feature space without loss in predictive performance.

**Figure 8:**
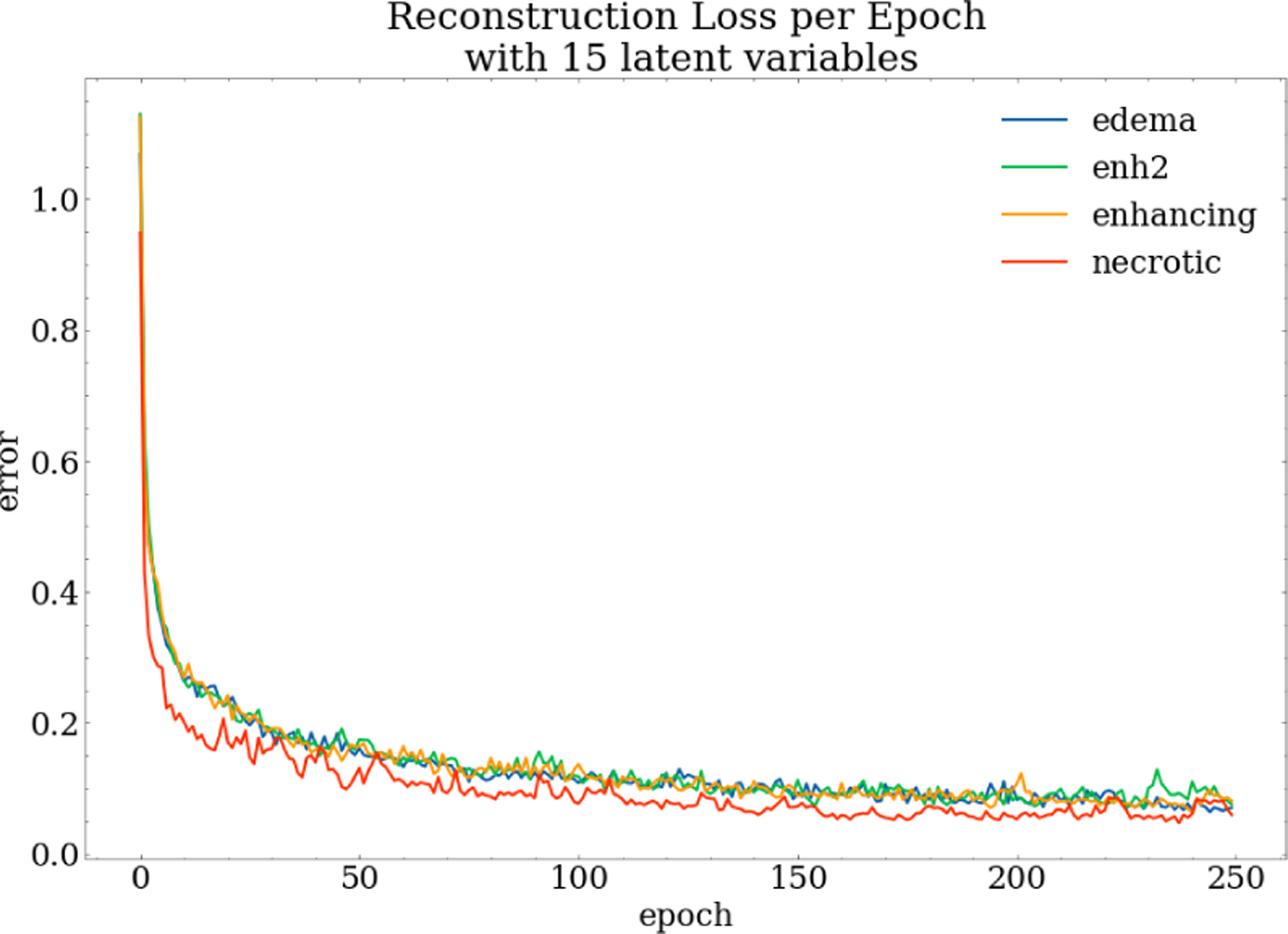
Autoencoder reconstruction error loss per training epoch for all ROI masks

Additional support that the autoencoders are iteratively learning the data representation for each of the masks is shown in Figure 8. The reconstruction error trend during training, i.e. the objective function, shows how well the model is training and can be informed if the correct learning rates are being used. Figure 8 confirms that the loss follows an exponential trend, minimizing below our threshold of < 0.2 of maximum reconstruction error indicating that the autoencoder is learning the representation well. Table 3 shows the 5×5-fold cross-validated AUCs, precision, recall, and F1-scores for the complete data set and three intervals of latent dimensions between 5 and 50.

The best-performing autoencoder used 15 latent variables per mask, resulting in a 97% decrease in dimensionality from 2,140 radiomic features to only 60 encoded features while maintaining comparable predictive performance to the full set. Moreover, performance between latent spaces in the range of 10–50 is comparable so the decision of best-performing autoencoder was a balance of performance and smallest dimensionality. All of the autoencoder AUCs match the performance achieved through dimensionality reduction with PCA in related works [26] and, with more investigation, have the potential to outperform other feature extraction techniques. In our prior work [26], the neural network model combined with PCA feature extraction only achieves an average AUC of 78% across individual sequences, compared to 91% AUC for the autoencoder approach.

#### 4.4.2 Two-Stage Architecture.

**Figure 9:**
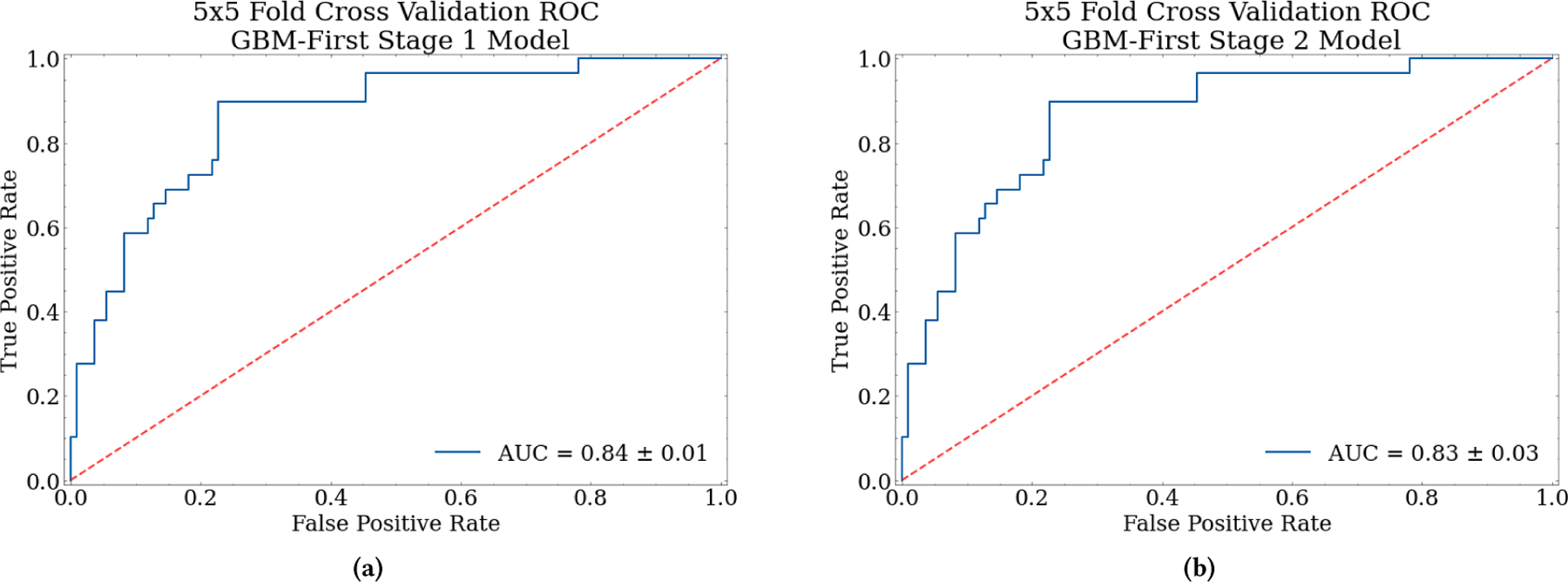
ROC AUC Curves for Two-Stage Architecture. The 1st stage classifier has primary class GBM and the combined secondary classes LYM and MET classified as “other”. The 2nd stage classifier has primary class LYM and secondary class MET with original labels. The dotted line indicates a random guess model.

An important decision in the two-stage architecture is the choice of the primary class for the first stage in the multiclass decomposition method. We chose GBM as the first classification task because it showed the best classification performance for the three-class results in terms of recall, precision, and F1-score. The first binary classification was defined as classifying GBM yes/no, where the label no corresponds to the combined MET and LYM samples. The second binary task was to distinguish MET from LYM; however, the largest class imbalance exists between the two. The LYM minority class samples were upsampled to have an equal proportion in the training data to MET. The second stage was tested on the true class label distributions. The individual performance of each stage was 0.83 and 0.84 AUCs shown in Figure 9, respectively. Table 3 also provides a summary of performance metrics for the two binary subtasks and the combined two-stage MLP results.

The performance metrics reported for the binary tasks consider only the class labels involved in training without propagating misclassified samples from the first stage into the second. The individual binary task performances show promising results with AUCs in the mid-80s. Each stage achieved an accuracy of 80% and 83%, respectively. The first-stage GBM classifier performed decently, though a stronger first-stage model is necessary for the success of our multiclass decomposition method. In related studies, AUCs for identifying GBM remain in the mid-90’s indicating that good results for the GBM classifier are possible. Still, more work is needed to match benchmark performance with GBM identification. This drop in performance could be due to the added complexity of combining the LYM and MET samples into one class, whereas previous studies had two distinct classes for comparison. The radiomic features for LYM and MET would theoretically have different patterns, so combining the samples into one “related” group could make it more difficult for the model to identify the patterns between the binary classes. While the problem is only partitioning two classes, there is still added complexity from the combination of LYM and MET rather than evaluating GBM and just one sample set.

**Figure 10:**
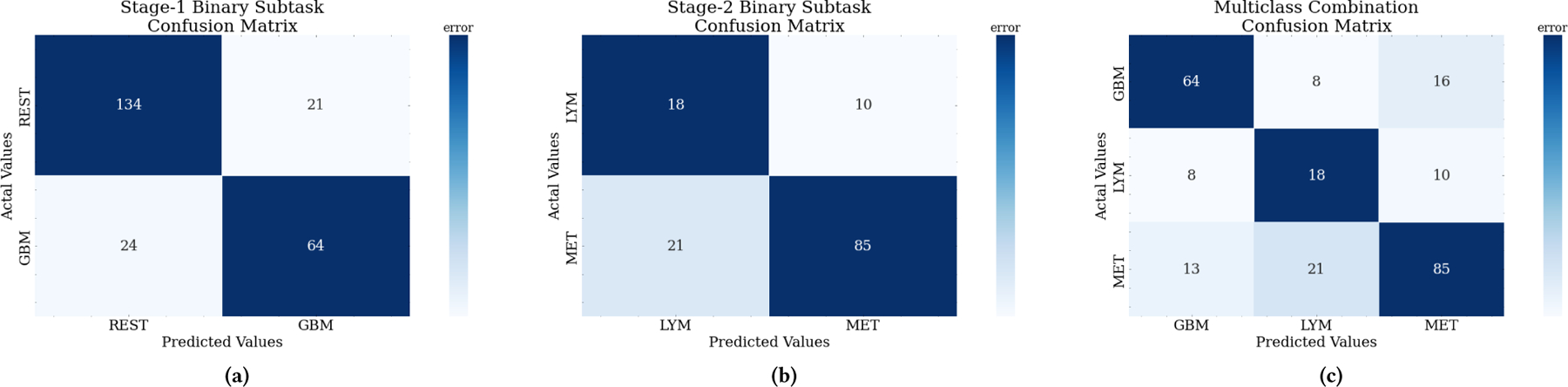
Confusion matrices for the actual vs. predicted tumor classes for both stages of the multiclass architecture as well as the composite multiclass performance. The diagonals in dark blue indicate correctly identified tumors and the off-diagonals are misclassified tumors.

The second stage classifier had improved performance from the first, achieving an AUC of 86% for distinguishing between LYM and MET cancers. This binary classifier showed promising results for identifying MET, but the model does not perform well on LYM, even with batch size adjustments and upsampling. The good MET performance argues for its use as the primary class for the first stage classifier rather than GBM. MET and LYM samples may be too imbalanced or too phenotypically similar to distinguish accurately alone. The class imbalance between GBM and LYM is lesser, and, as previously discussed, GBM and LYM have different tumor characteristics. Though, when considering the passed-through misclassifications from stage 1, the multiclass performance is less promising. The effects of this trickle-down misclassification can be understood through the confusion matrices of each of the stages as well as their combined performance as shown in Figure 10.

The first stage shows that 45 out of the 243 total samples are misclassified, with an accuracy of 81.4%. However, the 24 misclassified GBM samples will pass into the second stage during testing and be classified as either LYM or MET. Most of the remaining GBM samples are classified as MET in the second stage, as shown in (c). An almost equal proportion of METs were incorrectly classified as GBM in the first stage. It is worth noting that the biggest problem clinically is to distinguish GBM from MET as their appearances, irregular shapes and irregular contours, do overlap quite a bit.

The additive effect of the misclassification from each stage, and the inability to track back from the second stage and correct the misclassified labels motivates the need for a strong first-stage classifier.

## 5 CONCLUSION

The approaches to improve the preoperative characterization of malignant brain tumors via machine learning proposed in this work are twofold: reduce dimensionality by extracting non-linear patterns from radiomics and multiclass decomposition. The proposed methods leverage the feature learning capabilities of ANNs alongside the unsupervised nature of autoencoders to reduce the high-dimensional feature space of MRI-based radiomics. Furthermore, the multiclass decomposition approach leverages the promising performance of binary classifiers in clinical oncology by decomposing the multiclass problem into binary tumor identification problems and evaluating their composite performance.

The autoencoder feature learning approach effectively reduces the dimensionality by as much as 97% while maintaining the same performance as the full feature set with an AUC of 0.91. The autoencoders efficiently learned the representation of the tumor ROI masks with as few as 5 latent variables per mask, reaching peak performance with around 15 latent variables, with performance plateauing from there. We showed that when evaluating the performance of the autoencoder in each class, the overall accuracy and AUC for the minority classes LYM and GBM improved slightly.

Finally, the multiclass decomposition method showed modest results but provided ample motivation for future work. The composite multiclass performance was degraded by the misclassified samples in the first stage model. A stronger first-stage classifier with robust classification is necessary for the overall success of this approach.

Despite the performance degrading in the two-stage model, the proposed approach is still fairly promising at this stage for brain tumor diagnosis by domain scientists. A recent study evaluated the performance between human readers and human readers with the help of algorithms for a similar 3-class problem [33]. Even though the data size was much smaller, they noted that ‘two human readers achieved 65.4% and 80.8% accuracies, respectively. Using the MLP model as a computer-aided diagnosis (CADx) for cases in which the human reviewers disagreed with each other on the diagnosis resulted in correct diagnoses in 5 (19.2%) additional cases.’

A limitation of this work is that only MLP was used as the predictive model; however, many other models exist [6]. In related works, the random forest classifier outperformed MLP when using PCA for dimensionality reduction, using a similar MRI-based radiomics data set. Furthermore, the observable separability in the latent space mappings could suggest using other models like support vector machines (SVMs). Future work could focus on evaluating other models as well as training on additional data for improved performance using the encoded feature space. The feature selection steps prior to encoding also have the potential to boost performance; one could use correlation filtering [14] of the MRI-based radiomic features to remove the more redundant features prior to feature extraction.

In all, autoencoders have shown promise in the clinical oncology space for feature extraction on radiomics data. There are numerous potential directions and future work that can be applied to further test and improve their effectiveness for the classification of malignant brain cancers.

## Figures and Tables

**Table 1:** Malignant brain tumor class distribution.

		GBM	LYM	MET	Total
# Patients		93	40	120	253
% missing per mask	Edema	5.38%	10%	0.83%	3.95%
	Enc2	5.38%	10%	1.67%	4.35%
	Enhancing	5.38%	10%	0.83%	3.95%
	Necrotic	5.38%	85%	36.7%	32.8%

**Table 2:** Overview of hyperparameters tested in grid search for the MLP predictive models used and the best parameters chosen for each predictive task. Hidden layer sizes can be read as (#-nodes, #-layers) with ranges being listed for layers.

MLP Hyperparameters Tested		Best Parameters		
Parameters	Values	three-class	Stage 1	Stage 2
batch size	8,16,32,64,128	16	8	16
hidden layer	(50–200,1–5)*	(150,4)	(150,1)	(150,1)
learning rate	0.001, 0.0001	0.001	0.001	0.001
solver	adam, sgd	adam	adam	adam

* #-nodes are set as 50, 100, 150, 200.

**Table 3:** Model performance comparison. Average results for AUC, Precision (Pr), Recall (Rc), and F1-Score (F1) were computed using 5 times 5-Fold cross-validation for the two models. Performance for each class using micro-averaging (ALL).

Model	Setting	Class	AUC	Pr	Rc	F1
Three-Class MLP	Full set	GBM	.881	.830	.806	.818
		LYM	.859	.681	.705	.693
		MET	.920	.795	.803	.799
		ALL	.911	.790	.789	.789
	AE 5 latent	GBM	.894	.737	.778	.757
		LYM	.846	.667	.500	.571
		MET	.925	.800	.833	.816
		ALL	.887	.756	.760	.756
	AE 15 latent	GBM	.910	.801	.813	.807
		LYM	.897	.749	.640	.690
		MET	.923	.781	.810	.795
		ALL	.909	.783	.784	.783
	AE 50 latent	GBM	.910	.839	.806	.822
		LYM	.868	.639	.575	.605
		MET	.889	.776	.825	.800
		ALL	.899	.777	.779	.777
Two-Stage	Stage 1	GBM	.852	.828	.871	.849
		REST		.750	.682	.714
	Stage 2	LYM	.864	.667	.581	.621
		MET		.880	.913	.896
	Combined	ALL	-	.717	.712	.713

## CCS CONCEPTS

- Computing methodologies → Neural networks; Dimensionality reduction and manifold learning; Supervised learning by classification.

## Footnotes

1 The data is available for download at dx.doi.org/10.6084/m9.figshare.23599851
